# How Art Therapists Observe Mental Health Using Formal Elements in Art Products: Structure and Variation as Indicators for Balance and Adaptability

**DOI:** 10.3389/fpsyg.2018.01611

**Published:** 2018-09-05

**Authors:** Ingrid Pénzes, Susan van Hooren, Ditty Dokter, Giel Hutschemaekers

**Affiliations:** ^1^Faculty of Health Care, Department of Arts Therapies, Zuyd University of Applied Sciences, Heerlen, Netherlands; ^2^KenVaK Research Centre for the Arts Therapies and Psychomotricity, Heerlen, Netherlands; ^3^Faculty of Psychology and Educational Sciences, Open University of the Netherlands, Heerlen, Netherlands; ^4^Music and Performing Arts Department, MA Music Therapy and Drama Therapy, Anglia Ruskin University, Cambridge, United Kingdom; ^5^MA Dance and Music Therapy, Codarts University for the Arts, Rotterdam, Netherlands; ^6^School of Psychology, Radboud University, Nijmegen, Netherlands; ^7^Pro Persona, Centre for Mental Health Care, Nijmegen, Netherlands

**Keywords:** art therapy observation, formal elements, art product, adult mental health, qualitative study, grounded theory

## Abstract

In clinical practice, formal elements of art products are regularly used in art therapy observation to obtain insight into clients’ mental health and provide directions for further treatment. Due to the diversity of formal elements used in existing studies and the inconsistency in the interpretation, it is unclear which formal elements contribute to insight into clients’ mental health. In this qualitative study using Constructivist Grounded Theory, eight art therapists were interviewed in-depth to identify which formal elements they observe, how they describe mental health and how they associate formal elements with mental health. Findings of this study show that art therapists in this study observe the combination of movement, dynamic, contour and repetition (i.e., primary formal elements) with mixture of color, figuration and color saturation (i.e., secondary formal elements). Primary and secondary elements interacting together construct the structure and variation of the art product. Art therapists rarely interpret these formal elements in terms of symptoms or diagnosis. Instead, they use concepts such as balance and adaptability (i.e., self-management, openness, flexibility, and creativity). They associate balance, specifically being out of balance, with the severity of the clients’ problem and adaptability with clients’ strengths and resources. In the conclusion of the article we discuss the findings’ implications for practice and further research.

## Introduction

Formal elements of art products such as line, color and shape are often used in art therapy observation in youth as well as adult mental health care. The art therapists’ underlying assumption seems to be that formal elements reflect clients’ mental health problems (e.g., Cohen et al., 1986; Gantt and Tabone, 1998; Hacking, 1999; Conrad et al., 2011; Schoch et al., 2017). Observing formal elements could thus be used by art therapists to formulate their perspective on clients’ functioning, strengths and challenges and support their contribution to the descriptive diagnosis. This could help the art therapist to decide whether art therapy and which art interventions may be beneficial. This interest in the use of formal elements is reflected in a large number of studies (e.g., Elbing and Hacking, 2001; Stuhler-Bauer and Elbing, 2003; Betts, 2005, 2006; Mattson, 2009; Kim et al., 2012; Eytan and Elkis-Abuhoff, 2013; Thyme et al., 2013). These studies, however, demonstrate a wide range of opinions concerning which formal elements are relevant and how they are described and interpreted in art therapy observation and assessment. Also, prior studies used a different number of formal elements. In the diagnostic drawing series (DDS) (Cohen et al., 1986; Cohen, 1986/1994, unpublished) twenty-two formal elements are included. In the formal art therapy scale (FEATS) (Gantt and Tabone, 1998) fourteen formal elements are incorporated, in the descriptive assessment of psychiatric art (DAPA) (Hacking, 1999) five categories, and in the Nürtinger Rating Scale (NRS) (Elbing and Hacking, 2001; Stuhler-Bauer and Elbing, 2003) four categories of twenty-four formal elements. Even if similarities in these formal elements can be recognized, the way they are described differs largely. For example, regarding line some emphasize the quality of the line (Gantt and Tabone, 1998), whereas others emphasize the presence of line versus the absence of line (Cohen et al., 1986). Regarding color, the intensity of color is included in the DAPA (Hacking, 1999), whereas others include the mixture of color (Cohen et al., 1986). Additionally, diversity can be recognized in the methods used to observe and assess the formal elements. In some studies, open observation of formal elements is used to inquire into an in-depth understanding of the individual client (Stuhler-Bauer and Elbing, 2003; Thyme et al., 2013; Pénzes et al., 2015, McNiff in Gilroy et al., 2012). In other studies, specific assessment methods are used such as the DDS, in which an art therapist assesses three drawings that are made with colored pastels according to three different tasks, or the FEATS, in which a drawing made with markers is assessed.

The same kind of diversity is seen in the way formal elements are interpreted. In previous studies, formal elements are related to distinctive psychological features. In most studies, formal elements are related to disorders of the Diagnostic and Statistic Manual of Mental Disorders (DSM) or the International statistical classification of diseases and related health problems (ICD) (Cohen et al., 1986; Gantt and Tabone, 1998; Hacking, 1999; Kim et al., 2014). Whereas in more recent studies formal elements are related to clients’ strengths (Hinz, 2009, 2015; Pénzes et al., 2014, 2015) in line with perspectives on positive mental health (Huber et al., 2016) and recovery (Anthony, 1993). These perspectives have found their way into art therapy observation and assessment (Betts in Gilroy et al., 2012; Wilkinson and Chilton, 2013).

Thus, until now, literature has been far from consistent in presenting tools or suggestions directed toward the clinical use of formal elements in art therapy observation and assessment. Despite this ambiguous evidence, formal elements are very often used in clinical practice. Art therapists use existing art therapy assessment instruments in their own way, frequently developing their own assessment methods with their own favorite formal elements (Claessens et al., 2016). It is, however, unclear which formal elements art therapists find relevant in their clinical practice, how they observe and interpret them, and how art therapists relate formal elements to mental health. In this study, we will systematically investigate these aspects by interviewing art therapists with many years of experience in clinical practice. If indeed art therapists in clinical practice use formal elements in a consistent way, the outcomes of the present study may contribute to the ‘body of knowledge’ regarding if and how formal elements can be used in art therapy observation and assessment to estimate clients’ mental health, and direct further treatment.

## Materials and Methods

**FIGURE 1:**
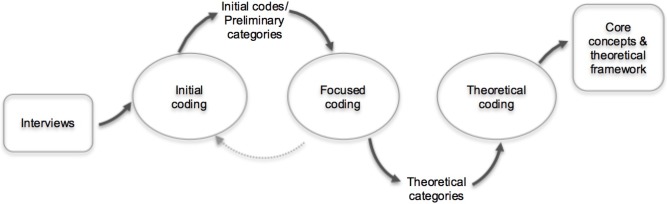
The research process.

In this study, we used Constructivist Grounded Theory (Charmaz, 2014). This qualitative approach inductively generates theory grounded in empirical data. Data was gathered through interviews with eight very experienced art therapists and analyzed by initial, focused and theoretical coding principles of qualitative analysis (Charmaz, 2014). See **Figure 1**.

### Participants

In total eight art therapists were purposely selected from the existing professional network of a research center of arts therapies in the Netherlands^1^. Participating art therapists were women with 15- >25 years experience with different populations and settings in adult mental health care. Based on the principle of theoretical sampling (Corbin and Strauss, 2008; Charmaz, 2014) they had different nationalities (Dutch, United States, and United Kingdom), diverse training backgrounds and art therapy perspectives. This diversity provided a critical exploration and variation of the concepts investigated in this study. All art therapists gave written informed consent in accordance with the Declaration of Helsinki.

### Data Collection

#### Art Products

The participating art therapists were asked to observe six art products of five clients with diverse mental health issues (see **Table 4**). Two art products (3 and 6) were made by one client. The art products were randomly selected from a larger sample of 138 products made by 48 clients of 11 art therapists. All clients gave written informed consent in accordance with the Declaration of Helsinki. All three paintings were made with acrylic paint on paper (size: 50 × 40 cm.) over a period of 3 weeks. For the first and second painting, the clients received standardized instructions to paint a landscape; for the third painting clients were asked to create a painting without instruction. For all paintings, clients received the same paint, color palette, brushes and pencils. The sampled clients had just started treatment in art therapy in a range of mental health settings. In this stage of treatment it is common in the Netherlands that the head of treatment (usually a psychologist or psychiatrist) formulates a preliminary DSM-diagnosis that might change during the course of assessment. Art therapists do not formulate a DSM-diagnosis. In a later phase they formulate an art therapy diagnosis and contribute to a general descriptive diagnosis.

#### Interviews

The art therapists were interviewed using “intensive interviews” (Charmaz, 2014). The aim of these interviews was to explore in detail which formal elements the art therapists observed and how they described mental health. In particular how exactly they related formal elements to what aspects of mental health. The first six interviews took place in the work setting of the art therapist. Interview seven and eight were conducted on Skype. All interviews were videotaped. During the live interviews the art products were spread out randomly. During the Skype interviews the art products were discussed in numerical order. The interviews were conducted with an interview guide based on the research questions of this study. First, every art therapist was asked to look at the art products separately, describe the formal elements and describe the first impression she gained about the client. Second, more general questions were asked about how she would define the concept of mental health. Finally, every art therapist was asked how she would relate the formal elements of the art product to various aspects of mental health.

This guide was used as a flexible structure to ensure detailed exploration of the art therapists’ view on formal elements of the art product, mental health and the interrelatedness of formal elements and mental health. Open and investigative questions (Charmaz, 2014) were asked to pinpoint these relationships in order to gain an understanding of the diagnostic value of formal elements of art products in art therapy observation; which formal elements *exactly* are important and how are they related to *exactly* which aspects of clients’ mental health?

### Data Analysis

#### Initial Coding

After full transcription of the interviews, text fragments were organized according to the topics of the interview guide. These topics, (1) formal elements, (2) mental health and (3) relationship between formal elements and mental health, were used as ‘sensitizing concepts’ (Charmaz, 2014). First, incident-by-incident coding took place; within each interview, each art product separately was analyzed. This analysis resulted in a set of codes.

#### Focused Coding

In the next stage these initial codes were further categorized by comparative analysis (see dotted line in **Figure 1**); input of all therapists and over all art products were compared and preliminary categories emerged. Codes about which most therapists agreed or that had similarities were clustered into theoretical codes regarding the formal elements, mental health and their interrelatedness. Based on the initial codes, these categories were described. Within the coding rocess, it became clear that some of these categories, such as several formal elements, were mentioned by almost all therapists about every art product; these became main theoretical categories. Some other categories were mentioned less often; these became sub theoretical categories. In this stage, a first perspective emerged on how the formal elements of the art product were related to mental health.

#### Theoretical Coding

**FIGURE 2:**
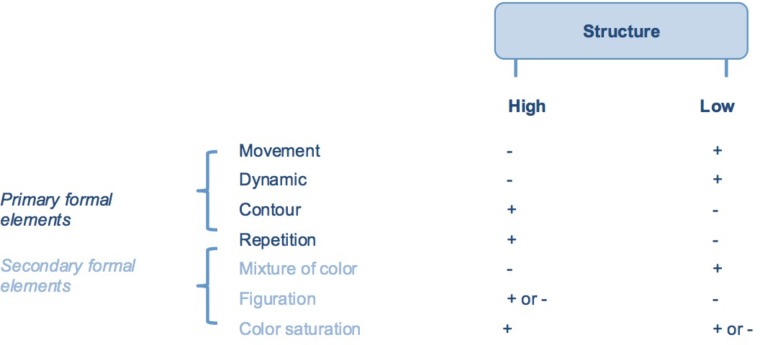
Combinations of formal elements determining the amount of structure.

Theoretical coding conceptualized the interrelatedness between the categories. Comparative analysis over the eight interviews supported synthesizing and organizing the links between the theoretical categories. Within this process some of the main theoretical categories were merged into core concepts. For example, the primary and secondary formal elements (see **Figure 2**) became main categories under the core concept “structure.” Of others, it became clear that they were related -but separated- main theoretical categories, such as flexibility and creativity under the core concept of “adaptability.” This led to a theoretical framework, conceptualizing the relatedness between sub- and main categories and core concepts. All were defined in detail.

### Quality

The whole process of analysis was peer debriefed to ensure that the codes, categories and concepts fit the data. The results of analysis were member checked with all art therapists on two occasions; (1) after initial coding; no additions or changes were made and (2) after focused coding; the art therapists gave some refinements and elaborations that were incorporated into the analysis.

## Results

### Formal Elements

Initial coding showed that art therapists used a variety of words (codes) related to the formal elements of the art products. Focused coding clustered these codes into seven categories (see **Table 1**). The formal elements “movement,” “dynamic,” “contour,” and “repetition” were mentioned most frequently. “Mixture of color,” “color saturation” and “figuration” were only mentioned by the art therapists when the element was dominantly present or absent within the art product.

**Table 1:** Description and illustration of formal elements.

Categories	Illustration by quotes and frequently used words	Description
**Formal elements**		
**Movement**	T2 (AP2): *“The movement is longer, fluent, continued and fairly monotonous.”* (AP 5): “*Coarse, pretty fast and short movements.”* Words with regard to the (1) amount: *a lot, little, more, less*, (2) character: *compact, short, long, round, straight, fluent, sketchy, small, large* and (3) Direction: *upward, horizontal, vertical, diagonal*	Movement refers to the amount, character and direction of the movement. Movement becomes visible by the brush marks.
**Dynamic**	T1 (AP5): *“There is a lot going on, it is very energetic and forceful. And at the same time it is somehow contained. By looking at the line and effect it, consciously or unconsciously, stopped.”* T4 (AP 4): *“I find this art product very turbulent. There is a lot of dynamic which gives me a restless impression.”* Words with regard to (1) A lot of dynamic: *lively, busy, forceful, energetic, powerful, turbulent*, and (2) Less dynamic: *static, rippling, reserved, contained, restrained, calm, timid*	Dynamic refers to tension (tectonic) within the art product. Dynamic varies between static, restrained and calm and fast, turbulent, energetic and forceful. It refers to the vitality of the movement made.
**Contour**	T8 (AP1): “*No fluidity, absolutely boxed-off; rigid distinctions, no overlapping, and more over; he keeps them [colors] very separate*.” T5 (AP2): *“In this case, the structure consists of the combination of movement and contour […].”* Words with regard to (1) A lot of contour: *delimitation, line marking, outline, blocks of colors, boxed off, rigid distinctions, sharp, straight lines*, and (2) Less contour: *fluent, overlap, impressionistic, pictorial, diffuse, loosely*	Contour refers to the delimitation that emerges when shapes are outlined or are placed straight next to each other. This leads to rigid distinctions.
**Repetition**	T4 (AP1): “*The repeated pattern of colors*.” (AP6): “*There is rhythm by the twist of the brush that is repeated throughout the art product.”* Words with regard to (1) the presence of repetition: *symmetry, mirroring, rhythm in movement, constancy, the same, pattern*, and (2*)* Absence of repetition: *no repetition*	Repetition refers to the return of one or more formal elements in a pattern. A high amount of repetition leads to symmetry. Rhythm refers to the repetition of movement.
**Mixture of color**	T8 (AP1): “*He is not mixing the colors, he is not playing with them, out of the bottle*.” Words with regard to (1) A lot of mixture: *mixed, tone, mix on palette/ paper, hue*, and (2) Absence of mixture: *straight from the bottle, separate, distinct, pure*	Mixture of color refers to the amount in which the colors are mixed within the art product.
**Color saturation**	T3 (AP4): “*The colors are not completely saturated*”. Words with regard to (1) High saturation: *covered, thick, impasto, opaque, filled in/up, texture, globs, dense*, and (2) Low saturation: *thin, dry, transparent, not covered, not filled, paper comes through*	Color saturation refers to the density of color within the art product varying between transparent and impasto.
**Figuration**	T6 (AP3): “*There is tried to make an actual image, positioning a tree in the foreground*.” Words with regard to (1) Presence of figuration: *figure, background, realistic, figurative, image, naturalistic, use of semantic color*, and (2) Absence of figuration: *abstract, basic, unrecognizable*	Figuration within the art product exists when there is tried to make a figurative or realistic image. Semantic use of color enhances figuration. Absence of figuration results in an abstract art product.
	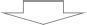	
**Structure**	T8 (AP4): “*There is organization, structure, absolutely. Painting the mountain and than the buildings*.” Related words: Gestalt, Overall Character Words with regard to (1) High structure: *organized, planned, sequence, layered, clear construction, controlled*, and (2) Low structure: *chaotic, unorganized, no structure, uncontrolled*	Structure refers to the way the art product is constructed and varies between clearly high and low structured. A highly structured art product is characterized as organized and planned. An art product with low or no structure is characterized as chaotic.
**Variation**	T2 (AP4): “*I see diverse movements; long, short, bended and different ways of using the paint; dry and wet. There is diversity and intention in this product.*” Words with regard to (1) Presence of variation: *diversity, differences, nuances, variety, divergence, differentiation, play*, and (2) Absence of variation: *no variation, limited, uniform, regular, equal, monotonous*	Variation refers to the diversity that can be recognized in one or more formal elements within the art product.

*T, therapist; AP, art product.*

#### Structure and Variation

The art therapists stated that formal elements might enhance or weaken each other and that *the combination* determined the “structure” of the art product (see **Table 1**). Structure varied between very high and very low structured.

Therapist 6: “I look for presence or absence of formal elements; which ones dominate? And how are they connected; the interplay determines the character of the art product.”
Therapist 5: “The structure of the art product consists of the interconnectedness of several formal elements and is indicative for how stuck a person is, how much space there is for change and that influences treatment.”

Focused and theoretical coding of all art products showed that highly structured art products consisted of the presence of “contour” and “repetition” (shown as “+” in **Figure 2**) in combination with the absence of “movement” and “dynamic” (shown as “-” in **Figure 2**). Absence of “mixture of color” and presence of “color saturation” enhanced the amount of structure. “Figuration” contributed sometimes to a highly structured product (e.g., art product 3), at other times not (e.g., art product 1).

Low structured art products consisted of the presence of “movement” and “dynamic” in combination with the absence of “contour” and “repetition.” Low structure was further weakened by the presence of “mixture of color” and absence of “figuration.” “Color saturation” contributed sometimes to a low structured art product (e.g., art product 5), at other times not (e.g., art product 1 or 2).

The presence or absence of “movement,” “dynamic,” “contour” and “repetition” determined the structure of the art product. These formal elements were mentioned most frequently. “Mixture of color,” “figuration” and “color saturation” reinforced or weakened this structure. To indicate the conciseness of “movement,” “dynamic,” “contour” and “repetition” in comparison with the other formal elements, the distinction between “primary” and “secondary” formal elements was introduced (see **Figure 2**).

How formal elements are combined and how strongly they are present appeared to determine the amount of structure art therapists perceived in the art product. The more dominant a formal element was present or absent, the more *clear the* structure of the art product was high or low.

The art therapists were in agreement about clearly structured art products. The dominant present or absent formal elements were consistently mentioned first. The art therapists were less consistent about art products that were less clearly structured. It seemed as if the art therapists hesitated and needed more time describing the formal elements. This was for example the case with art product 6 (see **Table 4**) that showed high structure but was far less structured than art product 1:

Therapist 2: “Somehow the others [art products] are more clear to me, here nothing jumps out. It is all the same, just the color is a bit different, and nothing is placed in the foreground. Those flowers feel a bit strange. It is not connected somehow.”
Therapist 5: “It has something threatening, almost as if it comes rolling toward me. It is a mountain, but it rolls in my direction. Probably accidentally painted in this manner…sort of. I don’t know…there are actually just four shapes with a cloud and some dots. Kind of duality, contradiction within the art product.”

Also, clear high or low structured art products demonstrated less *variation*. Variation emerged as a core concept by clustering categories related to the diversity within the art product (see **Table 1**). Variation existed when a range of formal elements was present or when there was diversity within one or more formal elements (e.g., diversity in movement by the presence of short and long, bended and straight lines). All therapists mentioned the amount of variation in each art product explicitly.

Therapist 8: About art product 1: “There is little variation; no mixing of colors, not playing with them, out of the bottle and more over he keeps them very separate. Lines are repetitive, no fluidity, absolutely boxed-off, rigid distinctions, no overlapping, juncture position of colors.”

### Mental Health

Anyone who expects that art therapists in these interviews used diagnostic terms such as depression and anxiety disorder in order to describe mental health will be disappointed. Art therapists were exceptionally reserved in using these terms and did not explicitly relate art products to psychopathology. However, art therapists did consider the art product as an important basis for clients’ mental state and consequently the possibilities and focus for treatment. Clients’ possibilities were mentioned more explicitly than their mental problems.

#### Clients’ Mental State: Balance

The art products provided cues to the art therapists about the clients’ inner world. They seemed to use an implicit conceptual model about the client’s balance. To describe this, they used a variety of terms. Most of them were related to “feeling” or “thought” (see **Table 2**).

**Table 2:** Categories of balance.

Category	Illustration by quote and frequently used words	Description
**Feeling**	T5 (Client 4): “*It is made very fast so I wonder if this client made this from a feeling or a memory? Does this client pay attention to how it felt to make this product? Does the client recognize to be in the “fast lane” often? So it is about feeling. I imagine this was made by a more disinhibited expressive person. It seems the client lost grip on itself a bit.”* *Being overwhelmed, feeling, lost in emotions, affective, not in control of emotions, uncontrolled, under regulation of emotions*	Feeling refers to affects and emotions and the ability to allow experience and express these. Feeling can be differentiated between “impulsive expression” (physically acting on feeling) and “emotional expression” (allowing and experiencing feeling).
**Thought**	T2 (Client 1): “*The need to control, shape raised from an idea, it is planned, thoughtful, restrained.”* *Ratio, cognitive, controlling emotions, need for predictability and structure, controlled, from the head, not feeling, analytical, thoughtful, over regulation of emotion, frantic, neurotic, planned.*	Thought refers to cognitions and cognitive processes that are related to cognitive control.

*T, therapist; AP, art product.*

With regard to *feeling*, all therapists mentioned emotion regulation and nearly all therapists focused on the client’s potential to regulate emotions. Art therapists extracted cues about feelings and emotions from the art products. Sometimes they referred to positive emotions (happy, lively), more often to negative emotions (anger, sadness, fear). It was as if they scanned the art product for cues with regard to the content of the emotion as well as the intensity of expression. Art therapists differentiated between clients who tended to express impulsively, i.e., physical acted on feeling, and clients that tended to express emotional, i.e., allowing and experiencing feeling.

Therapists 5: “Well, here [art product 4] it is about expressing feeling by using variation in color and movement and it seems to be about a memory or a story, whereas here [art product 5] it seems to be just about expressing emotions in an impulsive, more physical manner.”

Next, art therapists used words that could be related to *thought*. This category included thoughts, cognitions and cognitive processes such as planning, organizing, analyzing and structuring. Art therapists did not refer to the content of the cognitions but mostly to a continuum of cognitive control (see **Table 2**).

Balance existed when allowing, experiencing and expressing emotions and cognitive control were in proportion to each other. However, many clients showed themselves to be out of balance, either because of the high levels of emotion or because of the high levels of cognitive control.

#### Adaptability: Toward More Balance

Observation of a client’s tendency toward either “thought” or “feeling” enabled the art therapists to gain a perspective on clients’ actual balance. The art therapists stressed the importance to search for cues to estimate the client’s potential ability to achieve balance. This was related to clients’ “adaptability.” Adaptability was clustered into four categories; “self-management,” “flexibility,” “openness,” and “creativity” (see **Table 3**).

**Table 3:** Categories of adaptability.

Category	Illustration by quote and frequently used words	Description
**Self-management**	*T5: “Specifically that balance between emotion and ratio determines the ability to choose and self-determination. If someone only acts impulsive, that person is less able to decide.” Making choices, attention, self-determination, autonomy, identity, intention, position taking, and confidence.*	Self-management refers to the ability to choose. This requires the ability to distance and reflect, awareness of and paying attention to a present situation. Art therapists related self-management to self-determination, identity and autonomy.
**Flexibility**	T7: *“Someone that is mentally healthy is flexible: a person that is able and free to take in information through many means; body, mind and emotions, without blocks, obstacles and disconnections. Someone who is not mentally healthy is stuck in one way or not able to access or flexibly change between ways of processing*.” *Tuning, interact, being able to switch in response style adequate to a give situation, being able to adjust, resilient, integration of cognition and emotion, navigate, responsiveness to the situation, versatile* Absence of adaptability: *rigid, stuck, blocked, fixed*	Flexibility refers to the client’s range of possibilities to react to given challenges, tasks, persons or situations. This requires the ability to switch between cognitive control and allowing and expressing emotions. Therapists related flexibility to resiliency.
**Openness**	T5: “*Some sort of curiosity, openness, the ability to interact with the art material, to play with it, explore. Being open tells me something about the ability discover and learn.”* *Taking diverse perspectives, trying something new, experimenting, openness, not seeing a mistake as a disaster, exploring, differ from the known and familiar, taking risk*	Openness refers to an attitude that allows taking diverse and new perspectives. It involves curiosity, risk taking, not seeing mistakes as a disaster and daring to experiment in unfamiliar situations.
**Creativity**	*T1: “When someone wants to change, that means he has to transform and move from A to B. That requires leaving what’s familiar, expanding your horizon, facing and exploring the unknown.”* *Discovering, unconventional, combining things into something novel.*	Creativity refers to the possibility to differ from the known and leave beaten paths in order to create something novel. Art therapists related creativity to problem-solving.

*T, therapist; AP, art product.*

The combination of self-management, flexibility, openness and creativity determined clients’ adaptability. Art therapists were less positive about the adaptability of clients who were observed as struggling with being flexible, open, self-managed and creative (see **Table 4**).

**Table 4:** Art therapists’ observation of the art products and mental health.

Art product, instruction and preliminary diagnosis by psychiatrist or psychologist	Art product		Mental health according to the art therapists in this study		Formulated focus and duration of treatment

	Combination of formal elements - > structure	Variation	(Im)balance	Adaptability	
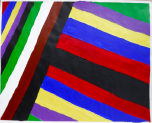 **Art product 1** **Instruction:** Painting free choice with acrylic paint; third session **Client 1:** Female, age 48, panic disorder, eating disorder (bulimia), depressive disorder, personality disorder	All therapists mention and agree on the clear high structure; *contour* and *repetition* are dominant present; the lines are placed straight to each other and the colors are, almost symmetrical, repeated. *Movement* and *dynamic* are dominate absent. The presence of *color saturation* and absence of *mixture of color* enhance contour and weaken movement and dynamic and enforce the high structure. Two therapists mention *figuration* (abstract).	All therapists mention and agree on the very limited variation; there is no differentiation.	Therapists find this client to be out of balance; very much *thought*; *cognitive control* and limited/ no feeling; restriction of allowing and expressing *emotions*.	This client is found to be the least adaptive (not *open* to experiment, not *flexible* because of the lack of variation and differentiation and not *creative* because of the need for predictability, yet some therapists mention that they find the art product powerful; a sort of statement related to *self-determination*). This client is described as anxious, neurotic and rigid. Overall, the therapists are not positive about treatment outcome and duration.	Therapists estimate that this client may benefit from affective experiences that allow access to and express feelings instead of controlling or avoiding them. However, they are cautious about how much the client needs the control of emotion as a defense. Therefore they expect more time may be needed in treatment.
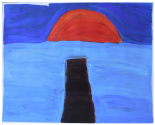 **Art product 2** **Instruction:** Painting a landscape with acrylic paint; first session **Client 2:** Female, age 20, Anorexia nervosa purging type	The therapists agree on the high structure; all of them mention the presence of *rhythm* (repetition of horizontal lines/ *movement)*, *high color saturation, figuration*, the calm *dynamic* and *contour* that is enhanced by limited *mixture of color* and *color saturation.*	All therapists mention and agree on the limited variation; monotonous and without differentiation.	Therapists find this client to be out of balance; having tendency toward *thought* and *cognitive control* out of fear to loose control over *emotions*. Three therapists find this art product a bit alarming by the lack of vitality.	This client is found to be limited adaptive (limited *open* as she seems to vary and experiment, limited *flexible* by the strong repetition, monotone movement and lack of differentiation, no *creativity* by the standard/ obvious and realistic figuration and limited yet some *self-determination* as the client seems to intentionally chooses this figuration and tries to work precise, yet not making the effort to correct “mistakes”).	To expand this client’s adaptability, the therapists prioritize stabilization and reinforcement of self-determination by enhancing affective experiences. The therapists are cautious positive about this client’s ability to change (she seems to be less stuck/ rigid as client 1 by the presence of some movement).
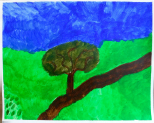 **Art product 3** **Instruction:** Painting a landscape with acrylic paint; first session	**Art product 3** The therapists agree on *figuration (*stylistic with no detail),* repetition* (rhythm of upward *movements*), *contour* (not dominate, yet present) and *dynamic* (seven therapists said to find it mechanically, one lively). Therefore the structure of this product is relatively high. Six therapists mention there is almost *no mixture of color*. Four therapists mention *color saturation* (varying but mainly transparent). These elements enhance the structure.	All therapists mention and agree there is some, yet restricted variation (a little bit more in art product 3 as in art product 4).	The therapists are less explicit and use diverse terms with regard to balance. In general this client is described as composed, tensed and tending toward *thought*; having the ability to regulate, but preferring to withhold from expressing *emotion*.	Therapists agree on this client’s adaptability (some *openness* by little experimentation but sticking to what is familiar, limited *flexibility* by lack of differentiation, no *creativity* by the very basic and obvious figuration which indicates no exploration and no diversion from the first idea, some *self-determination* by the choice of figuration, however, performed accidentally not seeing other possibilities).	The therapists are cautious positive about change; the limited amount of differentiation is seen as an opportunity to reinforce in treatment. However, some therapists question the client’s potential due to intellectual restrictions.
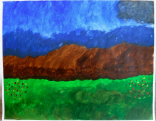 **Art product 6** **Instruction:** Painting a landscape with acrylic paint; second session **Client 3:** Female, age 33, Borderline Personality Disorder (main), depressive disorder recurrent moderate, problems related to upbringing children and work.	**Art product 6** Seven therapists immediately mention that art product 3 and 6 seems to be made by the same client. They agree that art product 6 tends to be highly structured, as *contour* is present by the distinction of the blocks of color. Yet there is some overlap. There is *repetition* by the rhythm of movement. *Movement* and *dynamic* are not completely absent. There is only little *mixture of color. Color saturation* varies between covered and dry. There is some *figuration*, mainly by the use of color. Six therapists have subjective associations, which are diverse but all related to the cloud.	
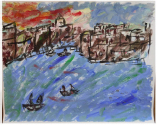 **Art product 4** **Instruction:** Painting a landscape with acrylic paint; first session **Client 4:** Female, age 48, Personality disorder (main), depressive disorder recurrent moderate.	All therapists mention the presence of *movement* and *dynamic* in combination with presence of *figuration* and some amount of *repetition*. Seven therapists mention *contour*, five mention *color saturation* varying between saturated and transparent/dry and four mention the presence of *mixture of color*. They agree that this art product is not completely chaotic or organized structured, yet slightly tending toward chaotic.	All therapists agree on the presence of variation.	Compared with the other clients in this study, the therapists find this client most balanced; despite having a tendency toward *feeling*, yet showing aspects of *thought*..	Therapists agree on this client’s adaptability (*open* as she seems to be experimenting and exploring, *flexible* as she seems to differentiate, *creative* as she seems not to be restricted to what is familiar or obvious, some limited *self-determination* as her impulses may block her from cognitive control.	Therapists are optimistic about change. They estimate that this client may benefit from cognitive experiences to develop the ability to stop and reflect and develop more cognitive control over feelings in order to enhance autonomy.
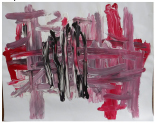 **Art product 5** **Instruction:** Painting free choice with acrylic paint; third session **Client 5:** Male, age 34, addiction (cocaine, alcohol) (main), dysthymic disorder, borderline personality disorder	All therapists agree on all formal elements and the rather low structure, even though it not completely lacks structure. *Movement* and *dynamic* (characterized as impulsive/forceful and contained/ restricted at the same time) are dominant present. These are enhanced by the presence of *color saturation* and *mixture of color*. *Contour* is not completely absent; the white paper creates distinction between the paper and the paint. Also *repetition* is present to some degree; horizontal and vertical lines are layered up, yet are less repetitive as the lines in art product 1.	All therapists agree on variation; limited present, all though the effect of paint seems to be repeated.	The therapists find this client out of balance toward *feeling* by the physically, yet not completely chaotic or impulsive, expression of feeling and some *cognitive control*.	Art therapists are moderate positive about this client’s adaptability (openness seems to be restricted by the repetition of the discovered effect of the paint, which may withhold this client from being *creative*, some *flexibility* by differentiation between expressing and structuring, presence of *self-determination*	Art therapists question this clients’ ability to change. They estimate that this client may benefit from experiences that expand the potential to regulate affect.

Therapist 7: “Persons that are willing in an unfamiliar situation, to put themself out, willing to learn and make mistakes and learn form their mistakes shows me something about their prognosis in therapy. Being able to face this task and adapt, that tells me something about their ability to learn and adapt in real life and that is a good prognosis.”

Art therapists in this study agreed that gaining a perspective on the client’s balance and the presence of adaptability gave direction to the formulation of treatment goals (see **Table 4**).

Therapist 2: “When someone over overregulates his emotions, tries to control them, treatment then is often focused on losing a bit of that control, being able to play, move and act and allow to feel. When someone under regulates his emotions, treatment is often focused on creating structure and calming down.”

### Formal Elements and Mental Health

**FIGURE 3:**
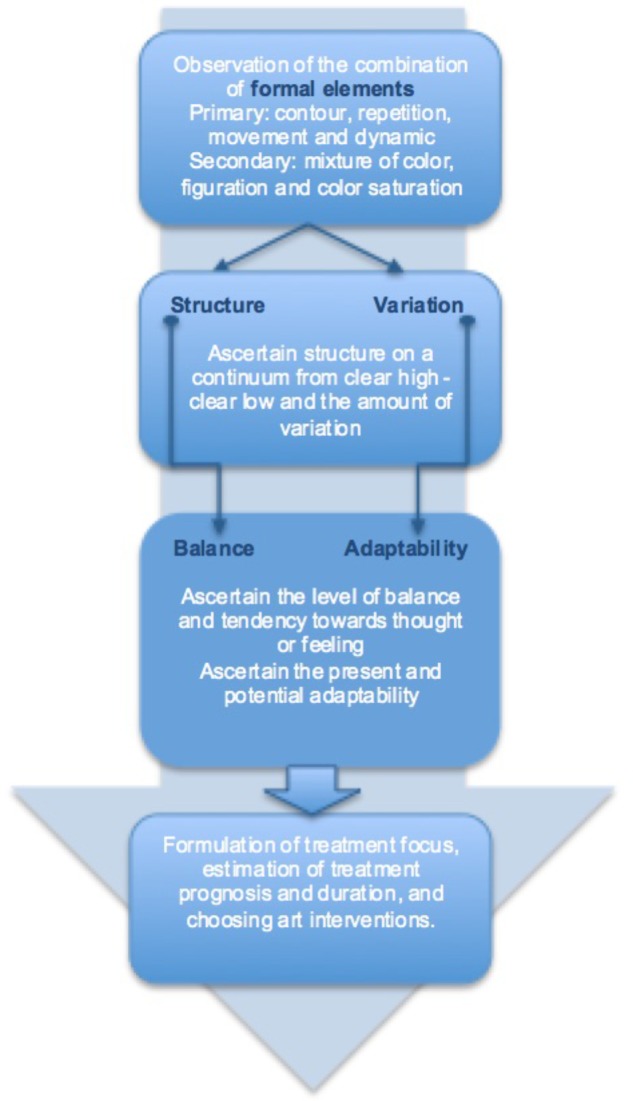
Formal elements in art therapy observation.

Through theoretical coding, relationships between formal elements and mental health were conceptualized in which the core concepts were related; structure was related to clients’ balance and variation was related to adaptability (see **Figure 3**).

#### Structure and Balance

Based on the observed primary and secondary formal elements, the extent to which they were present/absent and in which combination, art therapists ascertained the structure of the art product on a continuum from high to low. The structure was related to clients’ balance; based on one art product, art therapists were able to estimate the client’s balance. Art therapists preferred to observe at least three art products to observe if each art products had similar amounts of structure over time. If the structure of the art products did not change over time, their initial estimation of clients’ balance was confirmed. The art therapists assumed that art products with clear high or low structure indicated that the client was more out of balance. Three general patterns could be recognized; (1) highly structured art products were related to *much “thought” and less “feeling*”, (2) low structured art products were related to *more “feeling” and less “thought*” and (3) art products that alternated between high and low structure were related to *much “thought” and much “feeling”*.

#### Variation and Adaptability

Art therapists observed the amount of variation of the art product, which they related to “adaptability”. Variation was associated with experimentation, exploration, playfulness, taking risks, and discovery (see **Table 1**). These aspects were related to openness, self-management, flexibility and creativity; the categories of adaptability. Generally, more variation was related to more adaptability until a “turning point”. This means that art therapists assumed an “optimum” amount of variation; absent or limited variation as well as over- presence of variation was related to limited adaptability. Art therapists stressed their preference to observe at least three art products to observe variation within art products over time. Art therapists assumed that art products with limited or no variation over time indicated less adaptability. Variation over several art products indicated openness, willingness to learn and adapt. If variation was present over different art products art therapists were more optimistic about clients’ prognosis, as they associated adaptability with potential to change in therapy.

#### Balance, Adaptability and Further Treatment

The aim of treatment was to restore or develop balance between “thought” and “feeling” and to enhance adaptability. The art therapists in this study formulated the focus of treatment mainly on the estimated balance and potential adaptability. The focus of treatment directed the choice for art interventions. In general, the art therapists estimated that clients with a lot of cognitive control (i.e., thought) might benefit from more “affective” interventions, whereas clients with difficulties regulating their emotions might benefit from more “cognitive” interventions. Art therapists stressed the importance of the observation of clients’ individual position on the continuum of balance to choose the art interventions specific to the client’s needs.

Art therapists found clients’ balance and adaptability indicative for treatment duration and prognosis. They were more optimistic about the treatment duration and prognosis of clients who were more balanced and adaptive. Art therapists were alert to clients lack of balance and adaptability. They emphasized the importance of clients’ capacity to deal with change; they stated that a clear lack of balance and adaptability might have a function in survival and daily functioning. Therefore, treatment might take longer and prognosis, i.e., the expected amount of change, might be more limited.

## Discussion

Based on art products made with acrylic paint and the instruction to either paint a landscape or create a painting without instruction, all the art therapists in this study focus on four primary (movement, dynamic, contour, and repetition) and three secondary (mixture of color, figuration and color saturation) formal elements in art therapy observation. This implies that the art therapists in this study agree largely on the relevance of formal elements as well as their hierarchy. These seven formal elements show some resemblance to formal elements incorporated in existing studies on formal elements in art therapy e.g., “mixture of color” is also incorporated in the DDS, “color saturation” resembles “color intensity” of the DAPA, and “figuration” can be related to “color fit” of the FEATS. Some existing studies point out that individual elements mean nothing unless considered as a cluster (Gantt, 2001) or use a “profile” related to specific disorders, such as in the DDS. The findings of this study add to these studies by conceptualizing specific combinations of formal elements that construct the “structure” of the art product.

The art therapists in this study use the formal elements to estimate how clients make their art product. This is in line with existing studies in which it is theorized that formal elements reveal *how* the client makes the art product. It is this relation between formal elements and the making process that could explain why they provide information about clinically significant emotional and behavioral concerns of clients (Hinz, 2009; Conrad et al., 2011). In previous studies this was specified by the concept of material interaction (Pénzes et al., 2014, 2015).

With regard to the art therapists’ perspective on mental health, results of this study show that art therapists do estimate potential psychopathology. They rarely use symptoms or specific diagnoses used in DSM or ICD. Instead, they use concepts as balance and adaptability (i.e., self-management, openness, flexibility, and creativity). Emphasis on adaptability, i.e., resources and strengths, is in line with the perspective of positive health (Huber et al., 2016) in which health is defined as “the ability to adapt and self manage physical, emotional and social challenges in life.” This perspective shows resemblance to the “recovery approach”(Anthony, 1993), which focuses on fulfilling, meaningful life beyond the limitations of illness or symptomatology and emphasizes the empowerment of clients’ and their potential for change and growth.

The art therapists’ perspective on mental health certainly influences the way the formal elements are interpreted. Not relating the formal elements to symptoms and/or disorders, transcends any classification and is in line with other perspectives on mental health such as those of Siegel (Siegel, 2010, 2012, 2017) and Cozolino (2017) who also stress the importance of integrating “thought” and “feeling” to achieve, restore or maintain mental health and well-functioning.

If the art therapists observe clients being out of balance - which is more or less always the case in health care situations- they actively start searching for the elements of variation. Variation is associated with making choices, play, experimentation and exploration of the art materials. This relates to what in the literature is referred to as ‘material interaction’ (Pénzes et al., 2014, 2015). Material interaction refers to the clients’ dialog with art materials’ properties. Variation is subsequently related to self-management, openness, flexibility and creativity, i.e., adaptability. Hinz (2009); Lusebrink (2010), and Bucciarelli (2011) also pointed out creativity might be a sign of mental health, which emerged….however, they did not explore this further.

Balance and adaptability are conceptualized as two separate concepts; out of balance indicates the severity of the problems, whilst adaptability indicates present or potential resources which allow change in therapy. One might say that being out of balance refers to “mental-*illness*” and variation to “positive mental-*health.*” However, the therapists in this study often mention them together and point out that these concepts are closely interrelated, i.e., the severity of the problem and the potential resources of the client are seen as two sides of the same coin. This raises the question whether and to what degree balance and adaptability are independent, distinct, concepts. Literature on this matter is divided. Some studies question the distinction between mental illness and mental health (adaptation) (Lukat et al., 2016; Van Erp Taalman Kip and Hutschemaekers, 2018). The seven formal elements that emerged in this study might enable art therapists to gain perspective on the strengths and resources as challenges of clients. The findings add to studies that relate formal elements either to specific disorders or clients’ strengths and recourses in art therapy observation and assessment. The use of formal elements in art therapy observation provides a broader perspective on the client as a person.

### Critical Reflection and Implications for Practice and Future Research

This study conceptualizes three patterns of balance in combination with the variation in the art product. These patterns provide perspective on clients’ strengths, resources and challenges. It may be of interest in future research to investigate if and how the formal elements may differentiate in the way they are present between clients with diverse mental health issues.” Due to the limited number of art products included in this study, future research might include more art products to investigate if the same patterns emerge or if these patterns can be differentiated, specified or added.

Additionally, it may be useful to incorporate more than one art product of each client in future research to investigate if that leads to a more precise and differentiated observation of variation and adaptability. Even though all therapists largely agreed on clients’ variation and adaptability, they preferred more than one art product to estimate the variation.

Art therapists of three nationalities participated in this study. They cannot represent current international perspectives on art therapy assessment (Gilroy et al., 2012). However, they agreed on the formal elements and concepts of mental health. Findings of this study could be a valuable starting point to replicate the study in a broader international scope. Future research may address the potential of these concepts in contributing to the international current literature.

Existing studies on formal elements show that the formal elements observed are likely to change in response to the given task and the art media used. One could question if other formal elements would have emerged in this study when art products were made with a different task and art media. For example, the formal element “filled space” might have emerged when art products would have been made on larger paper size, with smaller brushes and/or allowing more time.

Nonetheless, the properties of acrylic paint, allow the observation of those formal elements which enable the art therapists to observe the structure and variation of the art product. The ability to observe the structure and variation related to the level of balance and adaptability may support art therapists to formulate treatment goals to suit the individual needs and potential and to choose those art interventions that enhance or develop the client’s balance and adaptability. Previous studies point out the therapeutic potential of diverse art material properties to achieve a more “affective” or “cognitive” experience to enhance “thought” or “feeling” (Hinz, 2009; Hyland Moon, 2010; Snir and Regev, 2013; Pénzes et al., 2014). The therapeutic value of experiential interventions is pointed out in many recent studies (e.g., Cozolino, 2017; Porges and Flores, 2017). Future research may address the use of art interventions to generate different affective or cognitive experiences.

## Conclusion

Formal elements are frequently used in clinical practice. In this study, we addressed two questions, namely which formal elements art therapists observe, and how they interpret them in terms of mental health. Findings add to the current body of knowledge. They show that the combination of seven formal elements construct the structure and variation of the art product and are indicative of clients’ level of balance and adaptability. Art therapists in this study gain insight into clients’ mental health through these concepts. This insight supports the art therapists in formulating treatment goals that suit the individual needs and potential and to choose those art interventions that ameliorate the client’s balance and adaptability.

## Ethics Statement

With regard to ethics approval, this study was conducted in 2016 and an ethics approval was not required as per our Institution’s guidelines and national regulations (Dutch “Law of medical research involving Human Subjects” [“Wet Medisch-wetenschappelijk Onderzoek (WMO)].”

The participants of this study consisted of art therapists, which were interviewed with regard to their professional method of observation of art products, their perspective on mental health and how they used the formal elements of the art product to gain insight in mental health. Prior to the interviews these art therapists were provided with written information with regard to the research aims and procedure. The interviews had duration of 1–1.5 h, in which the art therapists were interviewed on a familiar and professional topic. This research procedure was not considered as a risk of bringing the art therapist any possible harm. Written informed consent was obtained from all therapists in accordance with the Declaration of Helsinki.

With regard to the inclusion of the clients’ art products, written informed consent was obtained from all clients in accordance with the Declaration of Helsinki. Making art products with specific instructions as used in this study is common in clinical practice of art therapy and therefore considered as not harmful for clients.

## Author Contributions

IP developed the research design, conducted the research, and first authored this article. SvH, DD, and GH supervised the development of the research design and research process, and co-authored this article.

## Conflict of Interest Statement

The authors declare that the research was conducted in the absence of any commercial or financial relationships that could be construed as a potential conflict of interest.

## Footnotes

1 http://www.kenvak.nl/en/
